# Supplementary material for: Nanoimprinted and Anodized Templates for Large-Scale and Low-Cost Nanopatterning
Source: Nanomaterials (Basel). 2021 Dec 17;11(12):3430. doi: 10.3390/nano11123430 (PMC8707581; doi:10.3390/nano11123430)
Supplement: Supplementary file 1 [file nanomaterials-11-03430-s001.zip › nanomaterials-1503070-SI.pdf]

nanomaterials

Supplementary Information

Nanoimprinted and Anodized Templates for Large-Scale and Low-Cost Nanopatterning

David Navas *, David G. Trabada and Manuel Vázquez *

Instituto de Ciencia de Materiales de Madrid, ICMM-CSIC, 28049 Madrid, Spain; davidgonztra@gmail.com
* Correspondence: david.navas@csic.es (D.N.); mvazquez@icmm.csic.es (M.V.)

Table S1 collects the geometrical parameters, such as periodicity, P, linewidth, w, line height, h, and mold thickness, T, of the commercial optical disks employed as molds for the imprint step and with indication of whether single polycarbonate, PS, or polycarbonate stamp with metallic layer, PS/ML, were used.

Table S1. Geometrical parameters [1]: Polycarbonate section (Pol.): Polycarbonate stamp with metallic layer (PS/ML) or Polycarbonate stamp (PS); periodicity (P), linewidth (w), line height (h) and mold thickness (T).

Optical Disk	Pol.	P (nm)	W (nm)	h (nm)	T (mm)
**CD**	PS/ML	1600	600	130	0.1
**CD**	PS	1600	1000	130	1.1
**DVD**	PS/ML	740	320	65	0.6
**DVD**	PS	740	420	65	0.6
**BR**	PS/ML	320	130	40	1.1
**BR**	PS	320	190	40	0.1

SEM images of the PAA nanostructured templates, after a single anodization step of Al substrates and pre-patterned using a CD-PS/ML mold, are shown in Figure S1. PAAs with a single-imprint process exhibit two sets of parallel pores well aligned along the pre-patterning lines and separated by around 400 nm as well as around 800 nm width regions where the pores are randomly grown (see Figure S1 a and b). On the other hand, PAAs with a rhombic geometry also exhibit two sets of parallel pores well aligned along the pre-patterning lines, and a rhombic area with a lateral size of around 500 nm and where pores are mainly growing at the rhomboid edges (in Figure S1 c, d and e).

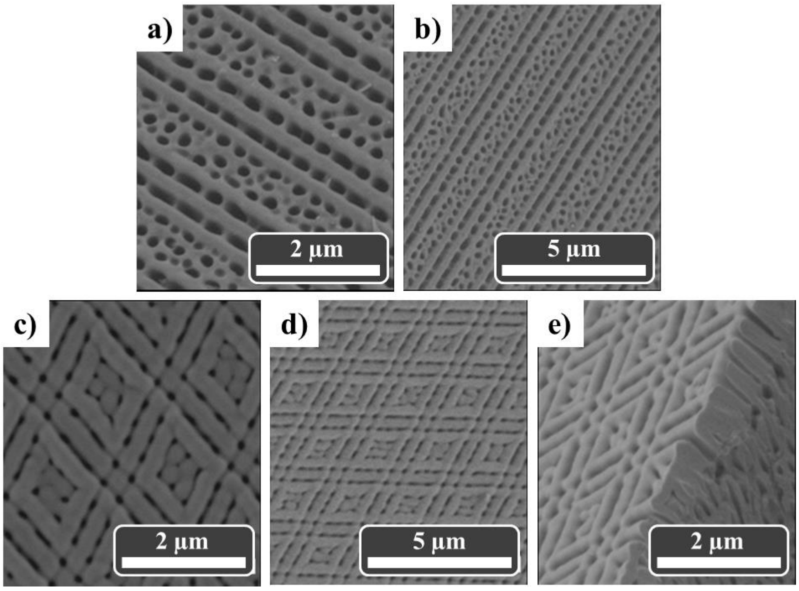

Figure S1. Top-view (a-d) and cross section (e) SEM images of PAA templates with line (a and b) and rhombic configurations (c, d and e) imprinted using CD-PS/ML molds and after a single anodization step under a constant voltage of 195 V in 0.03M H_3PO_4 acid solution at 5ºC and for 2 h.

Figure S2. depicts the SEM images of PAA nanostructured templates with line (a and b), square (c and d) and triangular (e and f) geometrical configurations imprinted using DVD-PS/ML molds and a single anodization step under constant voltage of 165 V in 0.03M H3PO4 solution at 5ºC and for 2 hours.

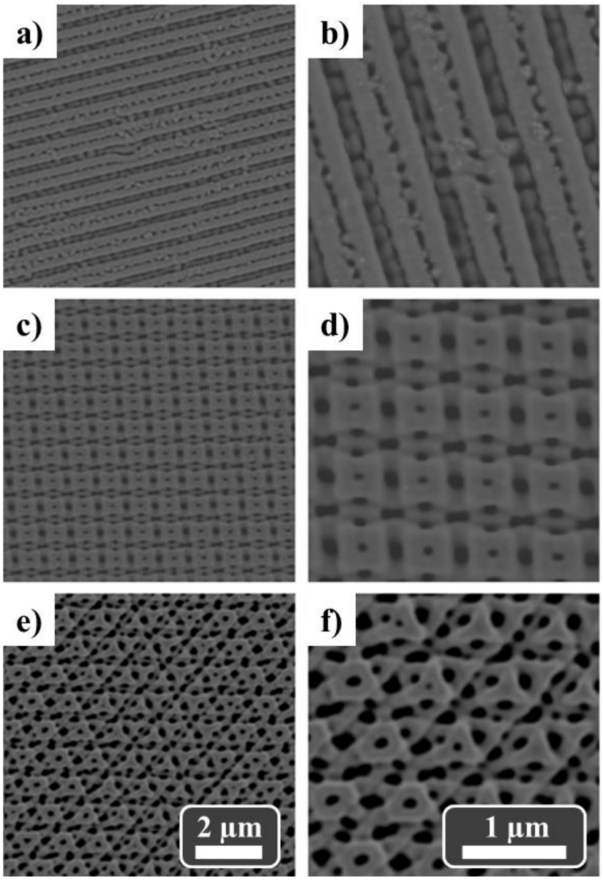

Figure S2. Top-view SEM images of PAA templates with line (a and b), square (c and d) and triangular configurations (e and f) imprinted using DVD-PS/ML molds and a single anodization step under constant voltage of 165 V in 0.03M H_3PO_4 solution at 5ºC and for 2 hours.

Figure S3 shows a free-standing square Co nanostructure after the chemical dissolution of the PAA template in a solution of phosphoric and chromic acids at 50ºC for 10 min.

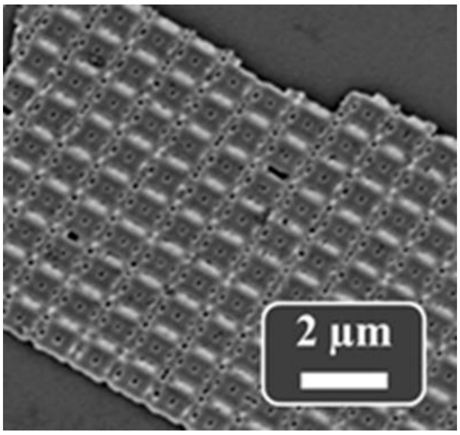

Figure S3. SEM image of a free-standing square Co-nanostructure.

Figure S4 shows the XRD patterns of the Co thin films sputtered onto the PAA templates.

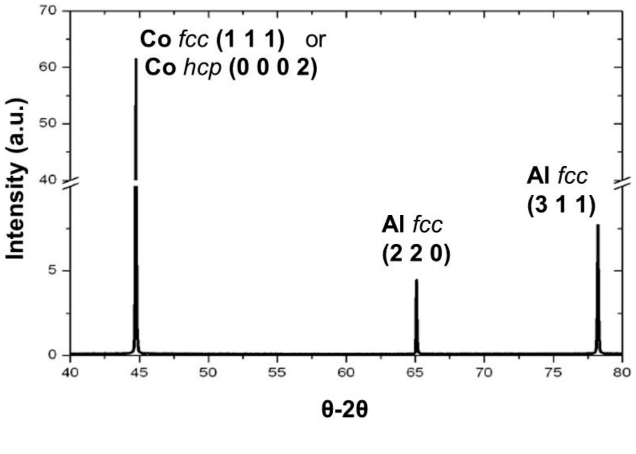

Figure S4. XRD pattern of Co nanostructures growth on PAA templates.

Figure S5 a shows the azimuthal or in-plane angular dependence of the VSM hysteresis loops for the Co nanodot array as well as its in-plane coercivity (Figure S 5b) and reduced remanence (Figure S5 c). In this analysis, the 0º orientation corresponds to the direction parallel to the side of the square dot. The hysteresis loops are clearly inclined with regards to the in-plane loops previously described for the arrays of Co nanostripes (see Figure 6 in the main text). In addition, the hysteresis loops do not show any significant evolution with the angular dependence which could be related to a less defined shape anisotropy term. A deeper analysis of the angular dependence of coercivity and remanence suggests the presence of a modest in-plane bi-axial magnetic anisotropy with magnetization easy axes at 90º to each other that can be correlated to the cubic array of squared Co nanodots.

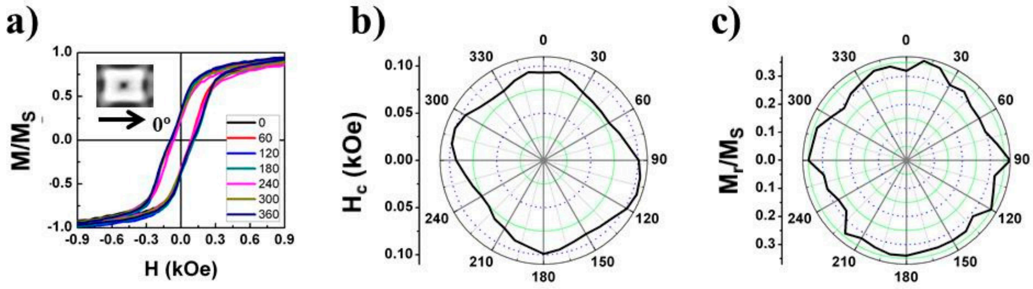

Figure S5. In-plane azimuthal VSM hysteresis loops (a) together with the corresponding dependence of coercivity, Hc, (b) and reduced remanence, M_r/M_s, (c) for a cubic array of squared Co nanodots.

References

S1. *http://osta.org/*
